# Group-based psychosocial interventions reduce internalized stigma in psychiatric disorders: ISMI-focused systematic review

**DOI:** 10.3389/fpsyt.2026.1724365

**Published:** 2026-05-20

**Authors:** Ioana-Alexandra Riviş, Sorin Ursoniu, Cătălina Giurgi-Oncu, Adriana-Camelia Neagu, Ion Papavă, Ana-Cristina Bredicean

**Affiliations:** 1 PhD School, Victor Babeş University of Medicine and Pharmacy, Timişoara, Timiş County, Romania; 2 Psychiatry and Safety Measures Hospital, Jebel, Timiş County, Romania; 3 Center for Cognitive Research in Neuropsychiatric Pathology (NeuroPsy-Cog), Victor Babeş University of Medicine and Pharmacy, Timişoara, Timiş County, Romania; 4 Department of Functional Sciences III, Discipline of Public Health and History of Medicine, Victor Babeş University of Medicine and Pharmacy, Timişoara, Timiş County, Romania; 5 Centre for Translational Research and Systems Medicine, Victor Babeş University of Medicine and Pharmacy, Timişoara, Timiş County, Romania; 6 Department of Neuroscience, Discipline of Psychiatry, Victor Babeş University of Medicine and Pharmacy, Timişoara, Timiş County, Romania; 7 Dr. Victor Popescu Military Emergency Clinical Hospital, Timişoara, Timiş County, Romania

**Keywords:** cognitive-behavioral therapy, group-based interventions, internalized stigma, ISMI scale, mental health recovery, psychiatric disorders, self-stigma, stigma reduction

## Abstract

**Background:**

Internalized stigma negatively impacts recovery outcomes, quality of life, and self-concept among individuals with psychiatric diagnoses. Group-based psychosocial interventions have been proposed as effective stigma-reduction strategies, but their impact across diverse populations remains under-evaluated.

**Objective:**

This systematic review synthesizes global evidence on the effectiveness of group-based interventions in reducing internalized stigma in adult psychiatric populations, with a focus on studies using the Internalized Stigma of Mental Illness (ISMI) scale.

**Methods:**

Following PRISMA 2020 guidelines, we searched PubMed, PsycINFO and Web of Science, and additionally screened full-text platforms (SpringerLink, ScienceDirect, SAGE Journals, and Wiley Online Library), for studies published between 2003 and 2025. Inclusion criteria required adult psychiatric populations, group-based interventions, and internalized stigma as a primary outcome. Study selection, risk of bias assessment, and data extraction were performed independently by two reviewers (US and GOC).

**Results:**

Ten studies [n= 1,088], across five countries, met inclusion criteria, including randomized controlled trials and pre-post designs. Most studies reported significant reductions in ISMI scores post-intervention, particularly in the domains of stereotype endorsement and social withdrawal. Culturally adapted interventions in China, Poland, and Spain demonstrated feasibility and impact, though subscale reliability varied regionally.

**Conclusion:**

Group-based psychosocial interventions may help reduce internalized stigma in psychiatric populations within an ISMI-based evidence base. The ISMI scale is, to this day, among the most frequently used instrument, though cultural adaptation of subscales such as stigma resistance remains a concern.

## 1 Introduction

Stigma associated with mental illness remains one of the most enduring and debilitating barriers to equitable mental health care. It reduces quality of life and impairs long-term outcomes by discouraging treatment engagement and reinforcing social marginalization (1–3). Stigma operates at multiple levels—public, structural, and internalized—each contributing to a climate of shame, alienation, and self-blame (4, 5). Internalized stigma is most concerning, as it represents a process by which individuals adopt and apply negative societal stereotypes to themselves, resulting in diminished self-esteem, reduced motivation, and social withdrawal (6, 7). Recognized as a transdiagnostic factor, internalized stigma adversely affects individuals across a range of psychiatric disorders, including mood disorders, schizophrenia, and personality disorders (8, 9). It is closely associated with delayed help-seeking, increased treatment dropout, and lower chances of psychosocial recovery (10).

Self-stigma, or internalized stigma, occurs intrapsychically and exerts a negative impact on identity, health behaviors, and self-concept (11, 12). Corrigan and colleagues proposed a four-stage model of self-stigma: awareness, agreement, application, and resulting harm to self-worth and self-efficacy (13). The culmination of this process, sometimes referred to as the “why try” effect, reflects a cognitive pattern in which individuals believe that efforts toward personal growth are futile (14, 15). Numerous studies have linked internalized stigma to poorer psychosocial outcomes, including lower self-esteem, increased depressive symptoms, reduced quality of life, and social isolation (5, 16). Particularly in individuals with severe mental illness, self-stigma may reinforce a “spoiled identity” that resists recovery without targeted psychosocial intervention (17).

Given these effects, intervention strategies that address both cognitive distortions and self-concept reconstruction emerged. Group-based psychosocial interventions—such as cognitive-behavioral therapy (CBT), narrative enhancement and cognitive therapy (NECT), and peer-led support groups—have demonstrated promise in reducing self-stigma and promoting empowerment. These approaches often incorporate cognitive reframing, social contact, and peer dialogue to facilitate identity reformation and stigma resistance.

The Internalized Stigma of Mental Illness (ISMI) scale, developed by Ritsher et al. (18, 19), is among the most frequently used instrument for measuring internalized stigma in clinical populations. The scale assesses five subdomains: alienation, stereotype endorsement, discrimination experience, social withdrawal, and stigma resistance. It has been psychometrically validated across various cultures with strong internal consistency (Cronbach’s α > 0.80) (20–22). However, cross-cultural reliability, particularly regarding stigma resistance, remains inconsistent. Cultural influences such as collectivism, religious framing of distress, and institutional legacies shape how stigma is internalized and reported (23, 24).

In Eastern Europe and the Balkans—regions characterized by post-communist transitions and entrenched institutional stigma—several studies have noted strong reliability for the ISMI overall, but low consistency for certain subscales, especially stigma resistance (e.g., α = 0.64 in Serbia) (25). In Romania, mental illness continues to be viewed through a lens of shame, silence, and moral judgment, resulting in elevated levels of internalized stigma (26, 27). Despite the ISMI’s use in Romanian samples—with good reliability overall—some subscales, such as stigma resistance and social withdrawal, require further adaptation to reflect culturally specific experiences (28). To the authors’ knowledge, to date, no empirical studies in Romania have evaluated the effectiveness of structured group interventions in reducing internalized stigma, despite international evidence suggesting their efficacy (29).

Studies conducted in Western and non-Western contexts offer convincing evidence for the effectiveness of group-based stigma-reduction interventions. A randomized trial by Yanos et al. (30) demonstrated that CBT-based group therapy significantly reduced ISMI scores in individuals with severe mental illness, particularly in stereotype endorsement and social withdrawal. The NECT model has shown similar results in Italy (31) and the Netherlands (32), including improvements in stigma resistance and hope. In Poland, Frączek-Cendrowska et al. (33) reported reductions in ISMI scores and increases in self-efficacy and coherence following CBT-based support groups. A systematic review by Jagan et al. (34) concluded that multimodal interventions integrating CBT, psychoeducation, and peer support are the most effective approaches to reducing internalized stigma. In China, Zhang et al. (35) adapted a group model for rural schizophrenia populations, reporting reductions in stigma and gains in empowerment. Similarly, Drapalski et al. (36) evaluated the “Ending Self-Stigma” (ESS) program across U.S. sites, showing improvements in ISMI outcomes and treatment engagement. These findings confirm the utility of structured group-based interventions, yet their effectiveness and cultural adaptability remain understudied in Eastern European contexts.

This systematic review aims to synthesize the existing evidence on the effectiveness of group-based psychosocial interventions targeting internalized stigma among adults with psychiatric disorders, with a specific focus on studies using the Internalized Stigma of Mental Illness (ISMI) scale as the principal outcome measure. This instrument-focused approach was chosen to improve comparability across studies, while recognizing that relevant evidence based on other validated self-stigma measures may fall outside the scope of the present review.

## 2 Methods

The review followed the Preferred Reporting Items for Systematic Reviews and Meta-Analyses (PRISMA) 2020 guidelines (37). No formal protocol was registered prior to conducting the review. The screening and selection process was carried out manually using a spreadsheet-based tracking system developed by the authors. Two independent reviewers (US and GOC) conducted title and abstract screening, followed by full-text eligibility assessment based on pre-defined inclusion criteria. Discrepancies were resolved through consensus discussion.

### 2.1 Data search

A systematic search was conducted across the electronic databases PubMed, PsycINFO, Web of Science from January 2003 to April 2025. This was supplemented by manual screening of publisher platforms (SpringerLink, ScienceDirect, SAGE Journals, and Wiley Online Library) and reference lists of eligible studies and relevant reviews. The lower bound of January 2003 was selected as it corresponds to the period in which the ISMI scale entered the literature and began to be used in intervention research. Given the central role of ISMI in the present review, this timeframe was chosen to capture the full period of relevant empirical work. The final database search was completed in April 2025. The search strategy was developed using combinations of keywords and subject headings related to internalized stigma, self-stigma, mental illness, psychiatric disorders, group-based interventions, support groups, CBT, and ISMI scale. Boolean operators were applied to enhance sensitivity and specificity. The search strategy combined free-text terms and, where appropriate, subject-related indexing terms referring to internalized stigma, psychiatric disorders, and group-based psychosocial interventions. The main search concepts included internalized stigma/self-stigma (“internalized stigma”, “internalized stigma”, “self-stigma”, “self stigma”), psychiatric populations (“mental illness”, “psychiatric disorder*”, schizophrenia, psychosis, depression, bipolar disorder, post-traumatic stress disorder, “severe mental illness”), intervention format (“group intervention”, “group-based intervention”, “group therapy”, psychotherapy, psychoeducation, “peer support”, “peer-led”, “support group*”, “narrative enhancement”, NECT, CBT, “cognitive behavioral therapy”), and outcome measurement (“ISMI”, “Internalized Stigma of Mental Illness”). These concepts were combined using the Boolean operator AND, while related terms within each concept were combined using OR. The syntax was adapted according to the indexing rules and search functions of each database. For example, in PubMed, combinations of title/abstract terms and controlled vocabulary were used where applicable, while in PsycINFO and Web of Science the strategy was adjusted to the specific subject indexing and topic-field structure of each platform. In addition, the reference lists of eligible articles and relevant review papers were screened manually in order to identify any further studies that met the inclusion criteria.

### 2.2 Eligibility criteria

Studies were included if they met the following criteria: (1) the publication was a primary full-text article available in English; (2) the sample consisted of adult participants (≥18 years) with a psychiatric diagnosis, as defined by DSM or ICD criteriano restriction was applied to a single diagnostic category at the eligibility stage; (3) the study implemented a group-based intervention (e.g., cognitive-behavioral therapy, psychoeducation, peer support, or narrative therapy); and (4) internalized stigma or self-stigma was assessed as a primary outcome, measured pre- and post-intervention using the ISMI scale, including full or modified versions.

Studies involving mixed populations (e.g., patients with both psychiatric and neurological conditions) were only included if outcomes for the psychiatric subgroup were reported separately. Articles that evaluated general stigma without reporting on internalized or self-stigma as a distinct construct were excluded.

### 2.3 Study selection

Two reviewers (US and GOC) independently screened the titles and abstracts of all identified records retrieved from the database search. Studies that met the inclusion criteria were then assessed at full-text level to determine final eligibility. Discrepancies during either stage were resolved through discussion and consensus. All decisions and records were tracked manually using a structured spreadsheet developed by the research team.

### 2.4 Data extraction

Data were independently extracted by two reviewers (US and GOC) using a standardized extraction template designed for this review. The following information was collected from each included study: author(s), year of publication, country of origin, study aim, research design, participant characteristics, diagnostic categories, description of the group-based intervention, comparison condition (if applicable), outcome measures used to assess internalized stigma (e.g., ISMI), time points of data collection, and primary results. Any discrepancies or uncertainties during data extraction were resolved through discussion and consensus.

### 2.5 Data quality

The methodological quality of the included studies was assessed using the Mixed Methods Appraisal Tool (MMAT), version 2018 (38). MMAT was selected because the included evidence base was methodologically heterogeneous and comprised randomized controlled trials, a quasi-experimental study, and an uncontrolled pre–post study. Although tools such as RoB 2 for randomized trials and ROBINS-I for non-randomized intervention studies are frequently recommended in systematic reviews, MMAT was considered the most appropriate single framework for consistent appraisal across the range of study designs included in the present review. This tool allows for the critical appraisal of studies across five distinct methodological categories: qualitative research, randomized controlled trials, non-randomized studies, quantitative descriptive studies, and mixed-methods research. Each included study was classified into its appropriate methodological domain, and five design-specific criteria were applied to evaluate potential sources of bias and methodological limitations.

Two reviewers (US and GOC) conducted the appraisal independently. Each criterion was scored as “Yes,” “No,” or “Can’t tell,” as per MMAT guidance. Discrepancies in ratings were resolved through consensus-based discussion. Following best practices, we did not compute an overall numeric score; instead, we reported each study’s performance across all five criteria to promote transparency and facilitate interpretation of methodological strengths and weaknesses. Accordingly, the MMAT appraisal was used to provide a structured overview of methodological rigor and potential sources of bias across studies, rather than to generate directly comparable risk-of-bias classifications across all designs.

This quality appraisal process allowed us to assess internal validity, risk of bias, and appropriateness of study designs within the context of stigma-related intervention research.

## 3 Results

### 3.1 Results of search strategy and study selection

The literature search across PubMed, PsycINFO, Web of Science, and full-text platforms such as SpringerLink, ScienceDirect, and Wiley Online Library yielded a total of 488 potentially relevant records. After removing 96 duplicates, 392 unique titles and abstracts were screened. Of these, 38 full-text articles were retrieved and assessed against the inclusion and exclusion criteria.

Based on this assessment, 10 primary studies met all eligibility criteria and were included in the final review. Studies were excluded at full-text review for reasons such as: ineligible intervention type (e.g., not group-based), absence of ISMI outcome measures, or protocol-only publications without results.

Due to heterogeneity in intervention formats, study designs, sample characteristics, and outcome reporting, a meta-analysis was not feasible. More specifically, the included studies differed substantially in several respects relevant to statistical pooling: study design (randomized controlled trials, quasi-experimental studies, and uncontrolled pre–post studies), intervention model (e.g., NECT, ESS, CBT-based, peer-led, and multimodal programs), comparator condition (treatment as usual, psychoeducational group, supportive group therapy, wait-list, matched controls, or no comparator), diagnostic composition of the samples, ISMI format used (29-item, 24-item, or versions excluding stigma resistance), and the way outcomes were reported across studies. In several cases, results were presented as within-group pre–post change, subgroup-specific effects, or interaction effects rather than as directly comparable between-group effect estimates with sufficient variance data for pooling. For these reasons, quantitative synthesis through meta-analysis was considered methodologically inappropriate, and a structured narrative synthesis was conducted instead.

**Figure 1:**
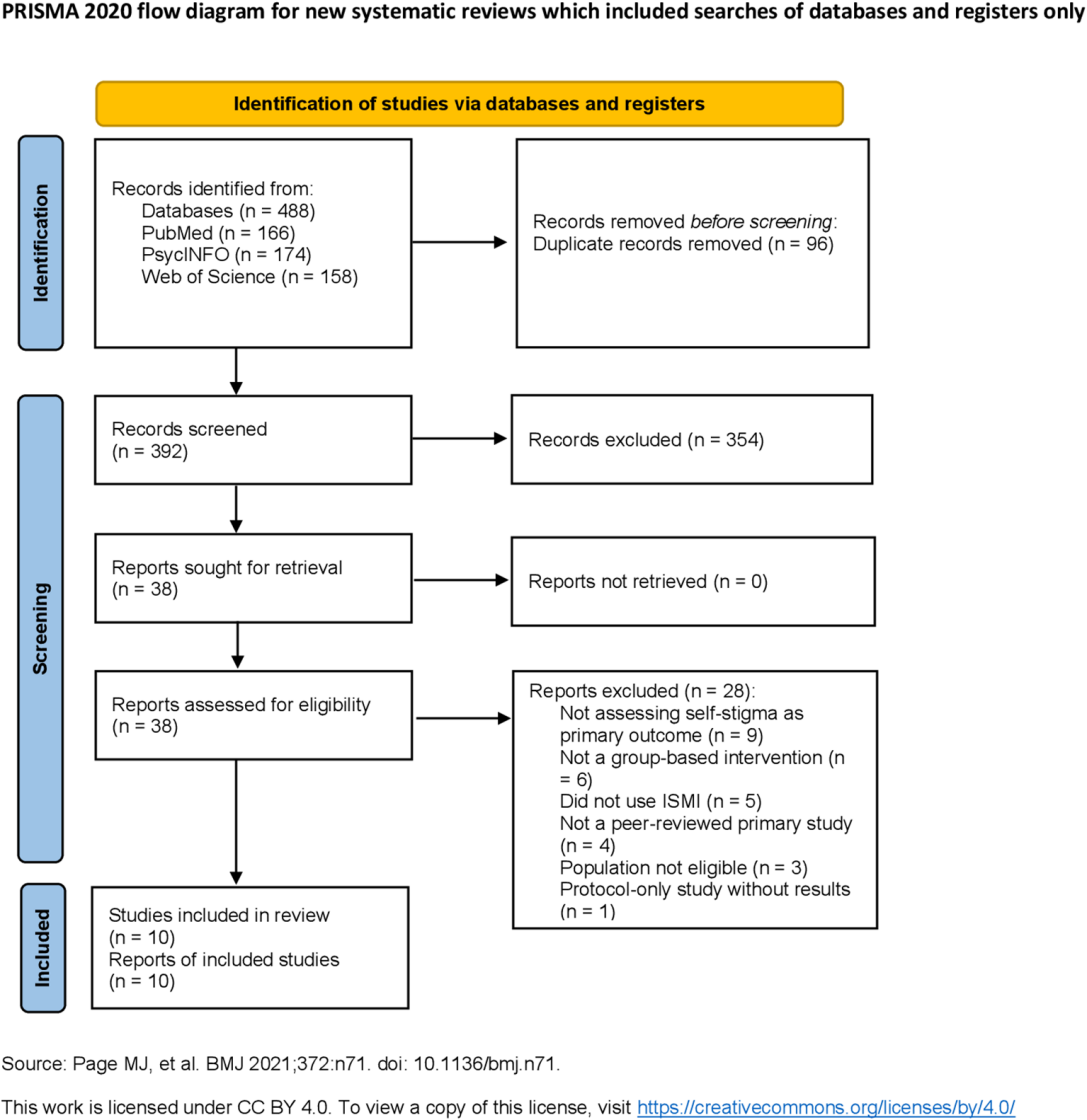
PRISMA 2020 flow diagram illustrating the article selection process. Source: Reproduced from Page et al. (37). Licensed under CC BY 4.0.

The screening and selection process is illustrated in the PRISMA flow diagram (**Figure 1**).

### 3.2 Characteristics of included studies

Ten studies were included in this review, published between 2011 and 2024, and conducted across five countries: the United States (n = 6), Poland (n = 1), Spain (n = 1), Israel (n = 1), and China (n = 1). The majority (n = 8) employed randomized controlled trial (RCT) designs, while one used a quasi-experimental design and one was a pre–post pilot study without a control group. Sample sizes ranged from 34 to 248 participants. Most participants were adults diagnosed with schizophrenia-spectrum disorders, though two studies included individuals with depression or post-traumatic stress disorder (PTSD).

All interventions were group-based psychosocial programs designed to reduce internalized stigma. The most commonly implemented intervention was Narrative Enhancement and Cognitive Therapy (NECT), used in three studies, followed by Ending Self-Stigma (ESS), used in two. Others applied structured, culturally adapted CBT-based protocols or multi-component approaches integrating psychoeducation, cognitive restructuring, and narrative techniques. The interventions ranged from 6 to 20 sessions, with weekly or biweekly delivery, and individual session durations between 60 and 90 minutes. Intervention facilitators typically included clinical psychologists, mental health professionals, or trained peer specialists.

Internalized stigma was measured using the Internalized Stigma of Mental Illness (ISMI) scale—either the full 29-item version or the 24-item version excluding the Stigma Resistance subscale.

**Table 1** provides a structured summary of the included studies, detailing country of origin, study design, sample size, psychiatric diagnoses, type of group-based intervention, comparator conditions, session frequency and duration, ISMI version used, and primary outcomes related to internalized stigma reduction.

**Table 1:** Characteristics of included studies examining group-based psychosocial interventions for internalized stigma in psychiatric populations.

Study (country)	Design	Sample (n)	Diagnosis	Intervention	Comparator	Sessions/duration	ISMI version	Main outcomes
Yanos et al., 2012 (USA) (30)	RCT (exploratory)	39 randomized	Schizophrenia, schizoaffective, bipolar, major depression	NECT	TAU	20 weekly sessions (phased)	ISMI (5 subscales)	Reduction in stereotype endorsement and increase in stigma resistance (trend-level) among exposed participants; no change in total ISMI
Frączek-Cendrowska et al., 2024 (Poland) (33)	RCT	104 randomized; 77 analyzed	Psychotic and affective disorders	“I am what I am” anti-stigma program	TAU	10 sessions, 60 min (2×/week)	Polish ISMI (5 subscales)	Significant reduction in total ISMI and subscales (alienation, stereotype endorsement, social withdrawal); no change in stigma resistance
Drapalski et al., 2020 (USA) (36)	RCT	248 randomized	Psychotic and affective disorders	ESS (Ending Self-Stigma)	Psychoeducational health group	9 weekly sessions, 75–90 min	ISMI-29	No group difference overall; significant ISMI reduction among participants with high psychotic symptoms
González-Domínguez et al., 2019 (Spain) (39)	RCT	80 (41 intervention, 39 control)	SMI	Multimodal group (CBT, ACT, narrative, peer storytelling)	Conventional treatment	9 weekly sessions, 90 min	Spanish ISMI (29-item)	Significant reduction in total ISMI and subscales (except stigma resistance); depressive symptoms also reduced
Russinova et al., 2014 (USA) (40)	RCT	82 (40 intervention, 42 control)	SMI (Axis I or II)	Peer-led Photovoice group	Wait-list control	10 weekly sessions	ISMI (5 subscales)	Significant reduction in total ISMI and stereotype endorsement; stigma resistance improved; other subscales unchanged
Yanos et al., 2019 (USA) (41)	RCT	170 randomized	Schizophrenia-spectrum disorders	NECT	Supportive Group Therapy	20 weekly sessions	ISMI (5 subscales)	Greater reduction in total ISMI, stereotype endorsement, and social withdrawal in NECT group at 3-month follow-up, especially in outpatient settings
Roe et al., 2014 (Israel) (42)	Quasi-experimental	137 NECT, 85 control	Psychiatric disability (SMI equivalent)	NECT	Matched regional control group	20 weekly sessions, 60 min	ISMI (excl. stigma resistance)	Significant improvement in alienation, stereotype endorsement, and social withdrawal; enhanced quality of life and self-esteem among NECT completers
Lucksted et al., 2011 (USA) (43)	Pre–post pilot study	34 analyzed	SMI	ESS (Ending Self-Stigma)	None	9 weekly sessions, 90 min	ISMI-29 and ISMI-24	Significant reduction in total ISMI and subscales; stigma resistance unchanged
Young et al., 2020 (Hong Kong) (44)	RCT (single-blind)	62 (31 per group)	Depression	CBT-based stigma group (culturally adapted)	Wait-list	10 weekly sessions, 90 min	Chinese ISMI-24	Significant reduction in total ISMI, moderate effect size; subscales not reported
Drapalski et al., 2021 (USA) (45)	Pilot RCT	57 randomized (24 analyzed)	PTSD (veterans)	ESS-P (adapted for PTSD)	Enhanced TAU	9 weekly sessions, 90 min	ISMI-24 (excl. stigma resistance)	Significant reduction in total ISMI: also improved self-efficacy and depression symptoms; no change in PTSD symptoms

### 3.3 Quality appraisal of included studies

Based on the MMAT assessment, three RCTs were rated as high quality (33, 41, 44), meeting all quality criteria including randomization, group comparability, complete outcome data, and strong adherence. Four RCTs were rated moderate quality (30, 36, 39, 40), primarily due to incomplete outcome data and lack of blinded outcome assessment. One further RCT (45) achieved a rating between moderate and high, as most criteria were met but outcome data were incomplete.

Given the methodological heterogeneity of the included evidence base, MMAT findings were interpreted at criterion level, with particular attention to randomization procedures, comparability of groups, completeness of outcome data, blinding, and intervention adherence in randomized trials, and to representativeness, validity of measurements, intervention fidelity, and outcome completeness in non-randomized designs. Overall, the appraisal suggested that the randomized studies generally showed stronger methodological rigor, whereas the non-randomized and uncontrolled studies were more vulnerable to incomplete outcome data and design-related limitations.

In Yanos et al. (30), for example, only 15 of 39 randomized participants attended ≥6 sessions, although intervention fidelity remained strong (mean fidelity = 4.4/5). Subgroup analyses suggested benefits among exposed participants (≥6 sessions), indicating that insufficient exposure rather than intervention inefficacy may explain weaker intent-to-treat results. Across RCTs, session adherence frequently exceeded 75%.

The two non-randomized studies were rated moderate quality. Roe et al. (42) employed a quasi-experimental design with strong intervention fidelity and valid measurement tools, but limitations included lack of randomization and attrition-related incomplete data. Lucksted et al. (43), a pre–post pilot without a control group, also demonstrated high fidelity and significant pre–post ISMI reductions, but its methodological strength was reduced by incomplete data handling and absence of a comparator arm.

Most studies utilized the full 29-item ISMI scale, though some excluded the Stigma Resistance subscale due to psychometric concerns. Even among high-quality studies, stigma resistance showed limited improvement, echoing broader concerns regarding its reliability across diverse populations.

Overall, intervention fidelity was consistently high, with structured manuals, trained facilitators, and standardized protocols. Feasibility was confirmed across diverse contexts, including U.S. VA clinics, Polish inpatient psychiatric units, and Hong Kong community centers, supporting the external validity of the findings. Nevertheless, attrition and the absence of blinded outcome assessment remain recurrent limitations.

A full summary of MMAT quality ratings by study is presented in **Table 2**.

**Table 2:** Quality appraisal of included studies using the mixed methods appraisal tool (MMAT, version 2018).

Study ID	Study design	MMAT 1 randomization?	MMAT 2 groups comparable?	MMAT 3 complete outcome data?	MMAT 4 blinded outcome assessment?	MMAT 5 adherence to intervention?	Overall quality
Randomized Controlled Trials							
Yanos et al., 2012 (30)	RCT	Yes	Yes	No	No	Yes	Moderate
Frączek-Cendrowska et al., 2024 (33)	RCT	Yes	Yes	Yes	Yes	Yes	High
Drapalski et al., 2021 (36)	RCT	Yes	Yes	No	Yes	Yes	Moderate
González-Domínguez et al., 2019 (39)	RCT	Yes	Yes	No	Yes	Yes	Moderate
Russinova et al., 2014 (40)	RCT	Yes	Yes	No	Yes	Yes	Moderate
Yanos et al., 2019 (41)	RCT	Yes	Yes	Yes	Yes	Yes	High
Young et al., 2020 (44)	RCT	Yes	Yes	Yes	Yes	Yes	High
Drapalski et al., 2021 (ESS-P) (45)	RCT	Yes	Yes	No	Yes	Yes	Moderate-High
Non-randomized and pre–post designs							
Study ID	Study Design	MMAT 1 Research question clear?	MMAT 2 Participants representative?	MMAT 3 Valid measurements?	MMAT 4 Intervention fidelity?	MMAT 5 Complete outcome data?	Overall Quality
Roe et al., 2014 (42)	Quasi-experimental	Yes	Yes	Yes	Yes	No	Moderate
Lucksted et al., 2011 (43)	Pre–post (no control)	Yes	Yes	Yes	Yes	No	Moderate

### 3.4 Results by outcomes

#### 3.4.1 ISMI total scores

Most interventions yielded significant reductions in total ISMI scores, with variations based on intervention format, population, and study design. A structured inpatient CBT-based program significantly reduced ISMI scores post-intervention compared to treatment as usual (*p* = .007, *η²* = 0.09) (33). A multimodal intervention in Spain combining CBT, ACT, and peer components also demonstrated a significant reduction in total ISMI scores (*p* ≤.01) (39).

Several U.S.-based trials supported these findings. A peer-led Photovoice program reported a moderate effect on overall ISMI reduction (*p* = .03, Cohen’s *d* = 0.55) (40). In a large pre–post pilot study, ISMI total scores decreased significantly (*t* = 3.59, *d* = 0.55, *p* <.01) (43). A culturally adapted CBT intervention in Hong Kong yielded significant pre–post change (*F* = 4.25, *p* = .04, *η*² = .10) (44). Among veterans with PTSD, a structured adaptation of ESS (ESS-P) also showed strong pre–post effects (*d* = 0.77, *p* = .007) (45).

Not all interventions demonstrated superiority over active comparators. A large randomized trial evaluating the original ESS intervention reported no significant differences between groups overall, but subgroup analysis showed significantly greater improvement for participants with high psychotic symptoms (*t* = –2.10, *p* = .036) (36). Similarly, the earliest exploratory RCT found no significant intent-to-treat effects; however, participants who attended ≥6 sessions reported trend-level improvements in total ISMI (30). In a more recent trial, total ISMI improvements were more pronounced among NECT participants in outpatient (low-intensity) settings, with a significant group × time × site interaction (*F* = 2.74, *p* <.05) and additional effects at three-month follow-up (*F* = 4.31, *p* <.05) (41).

A summary of the effects of group-based on total ISMI scores across the included study is presented in **Table 3**.

**Table 3:** Summary of effects of group-based interventions on total ISMI scores.

Study	ISMI total score outcome
Frączek-Cendrowska et al., 2024 (33)	↓ significant vs TAU (*p* = .007; η² = 0.09)
González-Domínguez et al., 2019 (39)	↓ significant post-intervention (*p* ≤.01)
Russinova et al., 2014 (40)	↓ moderate reduction (*p* = .03; *d* = 0.55)
Lucksted et al., 2011 (43)	↓ significant pre–post (*p* <.01; *d* = 0.55)
Young et al., 2020 (44)	↓ significant pre–post (*p* = .04; η² = .10)
Drapalski et al., 2021 (ESS-P) (45)	↓ strong pre–post effect (*d* = 0.77)
Drapalski et al., 2021 (ESS) (45)	↔ overall; ↓ in high psychotic symptoms
Yanos et al., 2012 (30)	↔ ITT; ↓ trend in exposed participants
Yanos et al., 2019 (41)	↓ greater effects in outpatient settings

Arrows indicate direction of effect on total ISMI scores: ↓ denotes a statistically significant reduction; ↔ denotes no statistically significant overall effect.

#### 3.4.2 Alienation

Alienation, reflecting feelings of exclusion and devaluation, improved significantly in multiple studies. A structured inpatient intervention reduced alienation scores compared to controls (*p* = .017) (33), and a pre–post study reported similarly strong effects (*t* = 3.33, *d* = 0.51, *p* <.01) (43). NECT demonstrated reductions in alienation in both a quasi-experimental study (42) and in exposed subgroups in an RCT (*F* = 2.98, *p* <.05) (41).

Other studies reported only trend-level or non-significant changes. In particular, the Photovoice intervention did not yield significant improvement in alienation (40), and no meaningful changes were detected in participants receiving the original ESS intervention (36).

#### 3.4.3 Stereotype endorsement

Stereotype endorsement showed consistent responsiveness across interventions. Significant reductions were observed in structured group CBT programs (*p* = .014) (33), and in multimodal programs (*p* ≤.01) (39). Peer-led interventions also performed well: Photovoice yielded a moderate, statistically significant effect (*p* = .05, *d* = 0.55) (40), while the pre–post pilot ESS intervention also reported positive change (*t* = 2.46, *d* = 0.43, *p* <.05) (43).

Further, one RCT demonstrated a significant group × time interaction for stereotype endorsement at three-month follow-up *(F* = 4.51, *p* <.05) (41). In another RCT, exposed participants (≥6 sessions) in NECT showed trend-level reductions (*p* ≈.097) (30).

#### 3.4.4 Discrimination experience

Evidence for reductions in perceived discrimination was mixed. Statistically significant improvements were reported in the quasi-experimental study of NECT (42), as well as in the pre–post ESS pilot (*t* = 2.40, *d* = 0.37, *p* <.05) (43). In an RCT of NECT vs. supportive therapy, a significant group × time × site interaction was detected, with stronger effects in outpatient settings (*F* = 3.41, *p* <.01) (41).

Conversely, other studies reported null findings. No change in discrimination experience was observed in the large Polish RCT (33) or in the peer-led Photovoice intervention (40). The multimodal intervention in Spain reported significant reductions in the “behavioral” component of stigma, which includes perceived discrimination (39), suggesting the need for broader conceptualization in future studies.

#### 3.4.5 Social withdrawal

Social withdrawal emerged as one of the most consistently improved domains. Significant reductions were reported in structured RCTs (33, 39, 41), in the quasi-experimental NECT evaluation (42), and in the pre–post ESS pilot (*t* = 3.35, *d* = 0.56, *p* <.01) (43). Notably, one trial showed strong group × time interactions both post-treatment and at three-month follow-up (*F* = 4.07; *F* = 5.26, *p* <.05) (41).

The Photovoice trial did not show significant change in this domain (40), and other studies such as ESS-P and NECT in earlier RCTs (30, 36, 44, 45) did not report subscale-level outcomes for social withdrawal.

#### 3.4.6 Stigma resistance

Stigma resistance was the least responsive domain across studies. A significant improvement was observed only in the Photovoice program, with the largest subscale effect size in that trial (*p* = .01, *d* = 0.67) (40). One exploratory RCT showed trend-level improvement in stigma resistance at three-month follow-up among exposed participants (*p* ≈.083) (30).

Most other studies reported no significant change (33, 39, 43), or excluded the stigma resistance subscale due to low internal consistency (44, 45). Although the full ISMI-29 was used in some studies (36), stigma resistance results were either unreported or non-significant, reflecting ongoing psychometric limitations in this subscale.

## 4 Discussion

### 4.1 Overview of intervention effects

Because the included evidence base comprised randomized controlled trials, a quasi-experimental study, and an uncontrolled pre–post study, the findings were interpreted with greater weight given to randomized comparisons, while results from non-randomized and uncontrolled designs were treated as supportive but less definitive. Accordingly, the overall conclusions of this review were based primarily on the direction and consistency of findings across the randomized trials, with additional contextual support drawn from the quasi-experimental and pre–post studies.

The reviewed studies suggest that group-based interventions may contribute to reductions in internalized stigma, particularly in domains such as stereotype endorsement and social withdrawal, although the magnitude and consistency of effects varied across intervention models and study designs. Across randomized and quasi-experimental trials, most interventions demonstrated improvements in total ISMI scores or subscale domains such as stereotype endorsement, alienation, and social withdrawal (30, 33, 36, 41, 42–43).

Multimodal programs integrating CBT with narrative and peer-led components—such as NECT (30, 41, 42), ESS (36, 43), and the Spanish hybrid program (39)—showed the broadest impact across ISMI domains. Reductions were especially notable for stereotype endorsement and social withdrawal, with several interventions yielding medium to large effect sizes (e.g., *d* = 0.55–0.67) (40, 43). However, stigma resistance remained largely unaffected in most trials (33, 36, 39, 43), suggesting that this construct may require more intensive or socio-politically grounded interventions to shift meaningfully.

The strongest support for benefit came from several randomized trials, although not all randomized comparisons demonstrated clear superiority over control conditions. Importantly, benefits were not always evident in intent-to-treat analyses; several studies found more substantial changes among participants who received adequate exposure (e.g., ≥6 sessions) (30, 41, 42). By contrast, findings from the quasi-experimental and uncontrolled pre–post studies were generally directionally consistent with the randomized evidence, but these designs are more vulnerable to bias and therefore should be interpreted as supportive rather than confirmatory.

Variability in treatment exposure and engagement may partly reflect broader beliefs about the value and effectiveness of psychotherapy. Recent work by Perricone and Ahn suggests that when mental illness is predominantly framed in biological terms, individuals may become less inclined to view psychotherapy as capable of facilitating meaningful change. Such biologically deterministic beliefs have been shown to reduce perceived effectiveness of psychological interventions, potentially undermining motivation, engagement, and sustained participation in treatment (46). In this context, the present findings indicate that structured group-based psychosocial interventions may have a corrective role by directly addressing maladaptive self-beliefs, reducing internalized stigma, and reinforcing psychosocial models of recovery within a biopsychosocial framework (40, 45–48).

### 4.2 Effectiveness by intervention model

Narrative Enhancement and NECT was evaluated in three high-quality studies. In the largest RCT, NECT outperformed a supportive group control in reducing self-stigma, particularly at 3-month follow-up and in outpatient settings (41). Improvements were significant for total ISMI scores, stereotype endorsement, and social withdrawal, with group × time effects consistently favoring NECT (41). Another quasi-experimental study reported similar domain-level benefits—especially in alienation and agency-related hope (42). However, an earlier trial showed only trend-level effects among participants exposed to ≥6 sessions (30).

ESS was supported by two trials. The large RCT found no overall between-group differences but showed that ESS significantly benefited participants with high psychotic symptoms (36). The pilot pre–post study reported consistent improvements across ISMI domains, including alienation (*d* = 0.51), stereotype endorsement (*d* = 0.43), discrimination (*d* = 0.37), and social withdrawal (*d* = 0.56) (43).

The CBT-based “I am what I am” program from Poland achieved significant improvements in total ISMI (*p* = .007), alienation, stereotype endorsement, and social withdrawal, with moderate effect sizes (*η*² = .06–.10) (33). This was one of the few trials to demonstrate strong adherence and high methodological quality on all MMAT dimensions except blinding.

The Photovoice peer-led intervention achieved statistically significant improvements in total ISMI (*p* = .03), stereotype endorsement (*p* = .05), and stigma resistance (*p* = .01) (40). The last of these was the strongest effect on stigma resistance observed across all studies (*d* = 0.67), underscoring the potential of empowerment-oriented formats to activate this otherwise resistant domain.

Lastly, a self-stigma CBT group for people with depression demonstrated positive effects in a culturally tailored RCT from Hong Kong, reducing ISMI total scores and subscale domains via restructuring culturally embedded stigma beliefs (44).

### 4.3 Intervention components and mechanisms

Most effective interventions shared core therapeutic ingredients: cognitive restructuring, psychoeducation, and narrative techniques. NECT’s structured blend of psychoeducation, challenging distorted thoughts, and identity reconstruction was particularly effective in outpatient, recovery-oriented settings (41, 42). Peer-led or peer-integrated models (e.g., Photovoice, Spanish hybrid model) may foster disclosure confidence and stigma resistance, although these effects were generally less consistent across studies. Some authors speculated that the stigma resistance subscale may not be responsive in the short term or lacks sensitivity (36, 39). The use of homework and real-life practice, particularly in the Spanish and Polish interventions, was cited as essential for generalization of gains beyond the group context (33, 39). These programs also emphasized internalization and real-world empowerment through structured sessions and progressive modules.

A further issue is the extent to which observed benefits were driven by specific psychotherapeutic techniques, such as cognitive restructuring or narrative work, versus the social contact inherent in group participation itself. In most included studies, these elements were delivered together within multicomponent group formats, making it difficult to isolate their independent contributions (30, 36, 39–43). Nevertheless, the group format likely functioned as more than a delivery platform: repeated contact with peers facing similar experiences may have reduced shame, normalized stigmatizing thoughts, and created opportunities for mutual recognition and social comparison. This interpretation is consistent with broader stigma-reduction literature, in which contact-based approaches are often more effective than education alone, particularly when contact is structured, emotionally meaningful, and recovery-oriented (6). Future trials would benefit from explicitly comparing psychotherapeutic content with social-contact-rich control conditions in order to clarify the relative contribution of these mechanisms.

### 4.4 Cross-cultural considerations

Cross-cultural context influenced how interventions were delivered, but also how self-stigma was conceptualized, internalized, and addressed within therapeutic settings. Despite using a common measurement framework (ISMI), the meaning and salience of stigma-related experiences varied across cultural and service environments.

Interventions implemented in Europe and Asia tended to be more structured and therapist-led, often delivered within inpatient or day treatment systems where participant engagement was built into clinical routines. These settings enabled high completion rates, but may have reduced opportunities for spontaneous peer exchange or identity reformation. In contrast, interventions conducted in North America more frequently occurred in community or outpatient settings, where attendance and engagement were more variable but recovery-oriented language and peer involvement were more central to the intervention ethos.

Cultural narratives about mental illness, identity, and disclosure also shaped the receptivity of participants to group-based interventions. In collectivist cultures, self-stigma may be more closely tied to family reputation and social obligation, necessitating adaptations in language and content to address shame and relational duty rather than solely internal belief systems. For example, interventions in East Asian settings included culturally embedded reframing strategies targeting self-worth in academic or filial roles, whereas Western programs emphasized individual agency and recovery.

Measurement sensitivity was also influenced by cultural framing. The stigma resistance subscale of the ISMI proved inconsistently responsive across sites—not necessarily due to intervention failure, but possibly because assertive resistance to stigma may be culturally discouraged, particularly in high-context, harmony-oriented societies. This may explain why resistance improvements were observed predominantly in empowerment-oriented, peer-led programs conducted in the U.S., and not in European or Asian settings where disclosure and resistance may carry higher relational or reputational costs (22, 23).

Taken together, these cross-cultural observations accentuate the need for flexible, context-sensitive intervention models that retain core components (e.g., cognitive restructuring, narrative work) but allow for cultural adaptation in tone, content, and facilitator style. Future work should explore how stigma manifests and is managed within specific cultural and systemic contexts, and how to design interventions that are both structurally robust and culturally congruent.

### 4.5 Implementation and feasibility

Most studies reported high adherence and feasibility. Interventions with manualized protocols and trained facilitators (e.g., NECT, ESS, “I am what I am”) showed consistent implementation (30, 33, 36, 39, 41, 43). High attendance (e.g., mean of 9.4 sessions in Poland, 13.8 in NECT exposed groups) suggests that stigma-focused interventions are acceptable in both inpatient and outpatient settings (30, 33, 41). However, dropout remained a concern, especially in longer interventions like NECT, where up to 28% attended fewer than four sessions (42). In some contexts, exposure to stigma content may itself provoke anxiety or withdrawal, particularly in early recovery phases (39, 42). Peer-led interventions like Photovoice were particularly well received but also required strong fidelity structures to ensure coherence and therapeutic safety (40).

An additional element warranting closer attention is the role of trained peer specialists. In several of the included interventions, especially peer-led or peer-integrated models such as Photovoice and the Spanish hybrid intervention, peer involvement may have contributed to both acceptability and the credibility of recovery-oriented messages within the group setting (39, 40). Peer specialists may strengthen identification, reduce hierarchical distance, and model stigma resistance in ways that differ from clinician-led psychoeducation alone. Recent evidence also suggests that peer support may be associated with improvements in personal recovery, hope, empowerment, and social inclusion, supporting its relevance as a complementary mechanism within anti-stigma interventions (47).

### 4.6 Methodological limitations

While several trials demonstrated high or moderate methodological quality, common limitations included incomplete outcome data, lack of assessor blinding, and short follow-up windows (30, 36, 40, 41). Nearly all studies relied on self-reported ISMI scores, and few included long-term assessments beyond 3–6 months. The use of active comparators in some RCTs (e.g., psychoeducation in ESS) may have diluted between-group effects (36). The stigma resistance subscale was inconsistently reported and often excluded due to psychometric concerns, limiting the ability to draw firm conclusions about this domain. Additionally, most trials did not assess qualitative change processes, session-specific effects, or broader functional outcomes like employment or quality of life.

### 4.7 Limitations

This review is subject to several limitations that warrant consideration. First, although a structured and transparent search strategy was applied, the scope was limited to peer-reviewed studies published in English, potentially excluding relevant findings from non-English language research or grey literature. This may have biased the dataset toward interventions tested in high-income countries with more formalized publication infrastructures. Second, the heterogeneity across studies in terms of design, intervention length, facilitation type, and outcome reporting limited the ability to conduct a meta-analysis or apply standardized effect size comparisons. While narrative synthesis allowed for the identification of broad patterns, differences in control conditions and ISMI subscale reporting made cross-study comparisons challenging. Also, several studies included in the review exhibited incomplete outcome reporting, lack of blinding, or short follow-up durations. These methodological constraints reduce the certainty of conclusions regarding the long-term impact and comparative effectiveness of interventions. An additional limitation is that the review protocol was not preregistered in PROSPERO or a comparable registry prior to study selection, which may reduce methodological transparency despite the structured review process employed. Another limitation is that the review was intentionally centered on ISMI-based outcome reporting. Although ISMI is one of the most frequently used and psychometrically examined self-stigma instruments in mental health research, other validated measures, including the Self-Stigma of Mental Illness Scale (SSMIS), were not the focus of the present synthesis. Consequently, the review should be interpreted as an ISMI-focused analysis rather than an exhaustive synthesis of all group-based interventions targeting self-stigma.

### 4.8 Future research directions

While the evidence base for group-based stigma interventions is growing, several gaps remain. Future studies should prioritize longitudinal designs to assess the durability of intervention effects, as current trials are limited by short follow-up periods. There is also a need for larger multisite RCTs that can test interventions across diverse clinical systems, populations, and cultural settings, enabling more generalizable conclusions. Methodologically, studies should incorporate mixed-method approaches, including qualitative interviews or session analysis, to better understand mechanisms of change and participants’ lived experiences. Given the limited responsiveness of certain ISMI subscales—particularly stigma resistance—future work may explore alternative or revised measures that are psychometrically robust and culturally sensitive. Finally, implementation research is warranted to guide the integration of these interventions into routine psychiatric care, particularly in low-resource and community-based settings where internalized stigma may be both more prevalent and more difficult to address.

## 5 Conclusions

This review suggests an emerging but still methodologically uneven evidence base for group-based interventions targeting internalized stigma in mental illness. Although the field has progressed beyond early proof-of-concept work, the current literature remains limited by heterogeneity in study design, intervention model, comparator condition, and outcome reporting. Across the included studies, several group-based interventions were associated with favorable effects on internalized stigma, supporting the view that self-stigma may be modifiable through structured psychosocial processes. Rather than focusing solely on symptom relief, these interventions address deeper constructs of self-worth, agency, and belonging. Their group format facilitates shared reflection, challenges internalized narratives, and often repositions participants as active contributors to one another’s recovery. At the same time, the present findings should be interpreted with caution, as the available evidence does not yet support strong conclusions regarding comparative effectiveness across intervention models, settings, or populations. Importantly, the review emphasizes that reducing self-stigma may have meaningful psychological and clinical relevance given its role in treatment engagement, social functioning, and long-term outcomes. At the same time, this body of evidence reminds us that stigma is culturally produced and contextually embedded. Interventions that appear successful in one setting may require thoughtful adaptation in another—not just in translation, but in how identity, illness, and resistance are understood. Thus, future efforts must balance fidelity with flexibility, ensuring interventions remain grounded in empirical evidence while resonating with the lived realities of diverse populations. Further well-designed studies, including prospectively registered trials with more consistent outcome reporting and longer follow-up, are needed before firmer practice recommendations can be made.

## Data Availability

The original contributions presented in the study are included in the article/Supplementary Material. Further inquiries can be directed to the corresponding author.
